# Lane Detection Based on ECBAM_ASPP Model

**DOI:** 10.3390/s24248098

**Published:** 2024-12-19

**Authors:** Xiang Gu, Qiwei Huang, Chaonan Du

**Affiliations:** 1 School of Artificial Intelligence and Computer Science, Nantong University, Nantong 226019, China; gu.x@ntu.edu.cn; 2 School of Information Science and Technology, Nantong University, Nantong 226019, China; 2330310006@stmail.ntu.edu.cn

**Keywords:** lane detection, autonomous driving, attention mechanism, deep learning

## Abstract

With the growing prominence of autonomous driving, the demand for accurate and efficient lane detection has increased significantly. Beyond ensuring accuracy, achieving high detection speed is crucial to maintaining real-time performance, stability, and safety. To address this challenge, this study proposes the ECBAM_ASPP model, which integrates the Efficient Convolutional Block Attention Module (ECBAM) with the Atrous Spatial Pyramid Pooling (ASPP) module. Building on traditional attention mechanisms, the ECBAM module employs dynamic convolution kernels to eliminate dimensionality reduction, enhancing the efficiency of feature channel learning and local interactions while preserving computational efficiency. The ECBAM_ASPP model incorporates the ECBAM attention mechanism into the feature extraction network, effectively directing the network to focus on salient features while suppressing irrelevant ones. Additionally, through variable sampling of the input, the model achieves multi-scale feature extraction, enabling it to capture richer lane-related feature information. Experimental results on the TuSimple and CULane datasets demonstrate that the ECBAM_ASPP model significantly improves real-time performance while maintaining high detection accuracy. Compared with baseline methods, the proposed model delivers superior overall performance, showcasing greater robustness and practicality.

## 1. Introduction

With the continuous development of the economy and society, the number of vehicles on the road has steadily increased. Consequently, various types of traffic accidents occur frequently, posing significant threats to human safety. Among the primary causes of these accidents are lane departures, highlighting the critical importance of automated lane localization in the domain of autonomous driving, with lane detection as a central focus. Lane detection serves as an effective method to guide vehicles within designated lanes, thereby mitigating the risk of lane departure incidents. This underscores the necessity for lane detection technology to achieve both high speed and precision.

Traditional lane detection algorithms often require extensive manual parameter tuning and exhibit poor generalization across varying scenarios. In recent years, deep learning-based approaches have become the predominant method for lane detection. Given that lanes possess high-level semantics associated with specific local patterns, accurate localization depends on detailed and robust feature extraction. However, effectively utilizing diverse and sufficiently rich features remains an open research challenge requiring further investigation. Lane detection is commonly modeled as a pixel-by-pixel classification task based on the concept of segmentation, where images are divided into lane regions and background areas. This approach employs supervised learning by integrating a lane instance discriminator into semantic segmentation models. For instance, earlier studies such as [1,2,3] utilized segmentation-based techniques to perform computations between dense bottom-up channels. However, these methods often suffer from high model complexity and suboptimal detection performance. To address these limitations, Zheng et al. [4] introduced the REcurrent Feature-Shift Aggregator (RESA) and the Bilateral Upsampling Decoder (BUSD) for lane detection. The RESA module enhances information transfer by periodically shifting slice feature maps both vertically and horizontally, effectively capturing pixel relationships across rows and columns. Similarly, Liu et al. [5] proposed CondLaneNet, a novel method that first detects the presence of lanes and dynamically predicts the shape of each lane instance when present. To handle the challenges of complex topological lane detection, their model incorporates the Ring Instance Module (RIM). Building upon these advancements, Wang et al. [6] developed the Global Association Network (GANet) and a lane-aware feature aggregator. This aggregator adaptively captures local correlations between neighboring keypoints while complementing global correlations with enriched local information. Collectively, these innovations tackle critical challenges in lane detection, advancing the field by improving accuracy and efficiency.

Although the above methods have improved the accuracy of lane detection, their computational complexity remains high, resulting in unsatisfactory detection speeds. Ensuring high detection speed, in addition to accuracy, is critical for maintaining real-time performance, stability, and safety. This is particularly important for autonomous driving and advanced driver assistance systems, where rapid adaptation to complex dynamic scenes is essential to deliver a reliable and seamless driving experience. Therefore, the primary objective of this study is to enhance lane detection speed without compromising accuracy. To address this challenge, we first aim to expand the receptive field of the model to enrich and refine the extracted features. Second, we prioritize focusing on regions of interest while effectively suppressing irrelevant areas. The main contributions of this paper are summarized as follows:

- In this paper, we propose the Efficient Convolutional Block Attention Module (ECBAM), which comprises two submodules: the Efficient Channel Attention Module (ECAM) and the Spatial Attention Module (SAM). ECBAM enhances the traditional Convolutional Block Attention Module (CBAM) by employing a dynamic one-dimensional convolution kernel in place of a shared Multi-Layer Perceptron (MLP).
- Building on the ECBAM and ASPP modules, this paper introduces the ECBAM_ASPP model. The ASPP module extracts multi-scale input features by applying different sampling rates, generating richer feature maps. These feature maps are combined with the attention weights generated by the ECBAM module to focus on lane areas while suppressing background interference.
- We evaluated the proposed ECBAM_ASPP model on the TuSimple and CULane datasets. The experimental results demonstrate that the ECBAM_ASPP model significantly enhances frames per second (FPS) while maintaining high accuracy. This indicates that the model ensures reliable lane line recognition while achieving high FPS, enabling vehicles to perceive road conditions in real time and make timely and accurate decisions.

## 2. Related Work

Developing safer and more reliable algorithmic mechanisms for advanced driver assistance systems (ADAS) is one of the primary objectives in the automotive industry. Lane detection, as a core component of these systems, plays a critical role in guiding vehicles to remain within designated lanes, ensuring safer navigation. Consequently, the real-time performance and accuracy of lane detection are subject to stringent requirements. Over the years, numerous studies have been conducted on lane detection, encompassing both traditional image processing methods and deep learning-based approaches. This subsection provides a brief review of key studies in the field of lane detection.

### 2.1. Lane Detection Methods Based on Conventional Image Processing

Building on traditional image processing techniques, Borkar et al. [7] proposed a lane detection approach that preprocesses the original image using an inverse perspective transform, applies a random sampling consistency algorithm to reduce image noise, and employs Kalman filtering to detect lane lines. Zheng et al. [8], Li et al. [9], and Wei et al. [10] utilized grayscale preprocessing and edge detection techniques, such as the Canny and Sobel operators, to extract edge feature information from lane images, followed by lane line detection using the Hough transform. Liu et al. [11] incorporated the brightness and width of lane markings, combining a local thresholding algorithm with the Hough transform and introducing auxiliary conditions to improve lane detection accuracy. Wang et al. [12] segmented binary features and converted them to an inverse perspective for fusion with inertial navigation system (INS) data from autonomous vehicles. After feature matching, parameter curves were fitted using the least squares method, followed by clustering analysis. Satzoda et al. [13] introduced an efficient hierarchical Hough transform (HT) framework, extending and applying additional properties of HT to multi-level hierarchies. Wang et al. [14] addressed challenges in lane positioning accuracy and control point failures by proposing a lane detection method based on non-uniform B-spline curve fitting, incorporating imaging model constraints to enhance robustness.

### 2.2. Lane Detection Methods Based on Deep Learning

Currently, lane detection methods have predominantly transitioned from traditional image processing techniques to deep learning approaches. In 2016, Wang et al. [15] introduced LaneNet, a lane detection method based on a deep neural network. LaneNet divides lane detection into two stages: lane edge extraction and lane line localization. Pan et al. [16] proposed the Spatial Convolutional Neural Network (SCNN), which replaces traditional layer-wise convolution in the vertical direction with slice-by-slice convolution on the feature map. This approach enables the transfer of feature information across rows and columns of pixels, enhancing spatial information propagation. Zhang et al. [17] developed a lane detection method based on a fully convolutional neural network (FCN). Their approach utilizes a compact neural network to efficiently extract lane line features from a large number of images. Qin et al. [18] proposed a novel formulation and structural loss function for explicitly modeling lane structures, reframing lane detection as a global feature-based lane line selection problem. Similarly, Zou et al. [19] designed a hybrid deep network combining convolutional neural networks (CNNs) with recurrent neural networks (RNNs) to account for the continuity of lane information across multi-frame images. Beyond the commonly used two-stage algorithms, Liu et al. [20] introduced an end-to-end approach that leverages Transformer-based networks to learn richer structural and contextual features. Their method directly outputs the parameters of the lane shape model, bypassing the need for intermediate steps. Pan et al. [21] propose an end-to-end lane detection method that employs a teaching–test module based on auxiliary supervision and knowledge distillation to directly predict the polynomial parameters of lanes. This teaching–test module guides the polynomial regression branch to learn shape features from the segmentation branch, enhancing fitting accuracy under complex road conditions. To address challenges such as lane occlusion and lane crossing, Chen et al. [22] proposed a Transformer-based dynamic kernel generation architecture for lane detection. This approach leverages a Transformer to generate dynamic convolution kernels for each lane line in the input image, which are subsequently used to detect these lane lines via dynamic convolution. Chai et al. [23] proposed a lane detection method based on row anchors, leveraging a Transformer encoder–decoder architecture. The row classification mechanism enhances the model’s capability to extract global features and detect lane lines in complex environments, enabling reliable lane detection in challenging scenarios.

### 2.3. Attention Mechanism

As a technique for enhancing feature extraction in deep convolutional networks, the essence of the attention mechanism lies in enabling the network to dynamically learn a series of weighting coefficients, thereby focusing on regions of interest. Hu et al. [24] proposed the Squeeze-and-Excitation Network (SE-Net), an effective channel attention mechanism that achieved significant performance improvements. Building on this, Li et al. [25] introduced Selective Kernel Networks (SK-Nets), inspired by the inception block and SE block. SK-Nets incorporate multiple convolutional kernel branches to learn attention across feature maps of different scales, allowing the network to emphasize significant scale features. Woo et al. [26] developed the Convolutional Block Attention Module (CBAM), a lightweight yet effective attention module for convolutional neural networks. CBAM sequentially computes attention maps for both channel and spatial dimensions of feature maps. It employs average pooling and max pooling to aggregate features in each dimension and multiplies the resulting attention maps with the input feature maps, achieving adaptive feature refinement. Wang et al. [27] introduced Efficient Channel Attention (ECA), which adopts a local cross-channel interaction strategy using 1D convolution to eliminate the need for dimensionality reduction, further enhancing efficiency.

## 3. Methodology

This section provides a detailed description of our approach, which integrates the Atrous Spatial Pyramid Pooling (ASPP) module [28], the Efficient Convolutional Block Attention Mechanism (ECBAM), and the proposed ECBAM_ASPP model.

### 3.1. Atrous Spatial Pyramid Pooling (ASPP)

Atrous Spatial Pyramid Pooling (ASPP) comprises a $\begin{array}{c} 1\times 1 \end{array}$ convolution, a pooled pyramid, and ASPP pooling. To enable flexible multi-scale feature extraction, the dilation rates of each layer within the pooled pyramid can be adjusted as required.

ASPP Convolution: The key distinction between dilated convolution and regular convolution lies in the dilation rate, which governs the spacing between kernel elements and the effective receptive field during the convolution process. By varying the dilation rates, different scales of receptive fields can be achieved, enabling the extraction of multi-scale feature information. However, the size of the convolutional kernel is consistently fixed at $\begin{array}{c} 3\times 3 \end{array}$.

ASPP Pooling: ASPP pooling is an adaptive technique, eliminating the need to specify kernel size and stride; only the final output size needs to be defined. It begins with an adaptive 2D average pooling layer, followed by adaptive mean pooling. Features are extracted by compressing the feature maps of each channel to a $\begin{array}{c} 1\times 1 \end{array}$ resolution to capture global features. Subsequently, the extracted features are refined and reduced in dimensionality using a $\begin{array}{c} 1\times 1 \end{array}$ convolutional layer. During forward propagation, the feature map is resampled back to its original size from the $\begin{array}{c} 1\times 1 \end{array}$ representation, ensuring that multi-scale global features are preserved.

### 3.2. Efficient Channel Attention (ECA)

The ECA module eliminates the fully connected layer used in the SE module and instead employs one-dimensional (1*D*) convolution on globally averaged pooled features to learn attention weights. The size of the 1*D* convolution kernel determines the number of channels considered when calculating each weight in the attention mechanism. ECA utilizes a fast, adaptive 1*D* convolution kernel of size *k*, which is proportional to the channel dimension and eliminates the need for manual adjustment. Here, *k* represents the extent of local cross-channel interactions.

Q. Wang [27] used the band matrix *Wk* to learn channel attention, and implemented a method for capturing local cross-channel interactions. The following is the *Wk* band matrix:

(1) $$\left[\begin{array}{c} \begin{array}{ccccccc} \omega^{1,1} & \cdots & \omega^{1,k} & 0 & 0 & \cdots & 0\\ 0 & \omega^{2,2} & \cdots & \omega^{2,k+1} & 0 & \cdots & 0\\ \vdots & \vdots & \ddots & \vdots & \vdots & \cdots & \vdots \\ 0 & 0 & \cdots & 0 & \omega^{C,C-k+1} & \cdots & \omega^{C,C} \end{array} \end{array}\right]$$

Appropriate cross-channel interactions enhance the transfer of feature information, thereby preserving model performance while reducing complexity. The local cross-channel interaction strategy in ECA, which operates without dimensionality reduction, avoids the adverse effects of dimensionality reduction on channel attention learning. This approach ensures more efficient and effective feature learning.

### 3.3. Efficient Convolution Block Attention Module (ECBAM)

#### 3.3.1. Convolutional Block Attention Module (CBAM)

The Convolutional Block Attention Module (CBAM) comprises two submodules: the Channel Attention Module (CAM), which performs channel attention, and the Spatial Attention Module (SAM), which performs spatial attention. The channel attention mechanism multiplies the input feature map by the corresponding elements of the channel weight matrix, followed by a feature recalibration process to produce a feature map that emphasizes key channel information. This recalibrated feature map serves as the input to the Spatial Attention Module, where it is further multiplied by the corresponding elements of two spatial weight coefficient matrices. The spatial attention mechanism adaptively weights spatial feature information through a sequential attention process, ultimately generating a feature map enriched with both channel and spatial positional information. These operations enable the network to focus on the most significant lane input features, enhancing spatial feature selection and improving feature representation.

#### 3.3.2. Efficient Convolution Block Attention Module (ECBAM)

In CBAM, the traditional channel attention mechanism treats each channel of the input feature map as a feature detector and compresses the spatial dimension of the input features. This involves a compression–expansion process for the channel dimension, which increases the model’s complexity. Inspired by the ECA strategy, which introduces a local cross-channel interaction mechanism without dimensionality reduction, we address the limitations of traditional channel attention by proposing ECBAM—an efficient and effective CBAM-based attention mechanism. ECBAM consists of two submodules: the Efficient Channel Attention Module (ECAM) and the Spatial Attention Module (SAM).

The main improvement of ECBAM is that it keeps the channel dimension constant and compresses the spatial dimension and it abandons the shared Multi-Layer Perceptron (MLP) module in learning channel features and directly uses $\begin{array}{c} 1\times 1 \end{array}$ convolution to learn the importance between different channels. We compress the entire feature maps in the spatial dimension and do not perform Global Max Pooling (GMP) and Global Average Pooling (GAP) in parallel on the input feature maps. At this point, the output dimension of our feature maps is still $\begin{array}{c} 1\times 1\times C \end{array}$, avoiding dimensionality reduction.

The size of the convolution kernel affects the sense field when operating with $\begin{array}{c} 1\times 1 \end{array}$ convolution. We use a $\begin{array}{c} 1\times 1 \end{array}$ dynamic convolutional kernel to improve the adaptability of the feature extraction and to solve the problem of mapping different input features. To accurately capture local cross-channel information interactions, it is necessary to determine the approximate range of channel interaction information (i.e., the size of the convolution kernel *k* for a one-dimensional convolution). There can be a mapping relation ϕ between *k* and *C*, C=ϕ(k). Namely, *k* is proportional to the channel dimension *C*. In neural networks, the channel dimension *C* (number of filters) is usually set to a power of two. Based on this, we extend the linear function to a non-linear function $C=\phi \left(k\right)=2^{\left(\gamma *k-b\right)}$, which implements the adaptive determination of the kernel size *k* based on the channel dimension *C*. The following equation determines the size of the convolution kernel *k*.

(2) $$k=\phi \left(C\right)={\left|\frac{\log_{2}\left(C\right)}{\gamma }+\frac{b}{\gamma }\right|}_{odd}$$

where *k* is the size of the convolution kernel and *C* is the number of channels. ${\left|\right|}_{odd}$ means *k* can only be an odd number. In this paper, we set γ and *b* is 2 and 1 to change the ratio between the number of channels C and the size of the convolution kernel. Meanwhile, we make all channels share the same learning parameters to reduce the number of parameters and improve the performance of the model. This can be easily achieved by a fast 1*D* convolution with kernel size *k*, that is, based on Equation (2), Equation (3) is added as a constraint, where *C*1*D* stands for 1*D* convolution. The formula is as follows:

(3) $$\omega =\sigma (C1D_{k}\left(F\right))$$

After 1*D* convolution, the sigmoid function obtains the channel attention information. Then, the feature map containing the channel attention information ($\begin{array}{c} 1\times 1\times C \end{array}$) is multiplied channel by channel by the original input feature map ($\begin{array}{c} H\times W\times C \end{array}$) to obtain the final channel attention feature map. In this way, the ECAM is calculated as follows:

(4) $$EM_{c}\left(F\right)=\sigma \left(ClD_{k}\left(GAP\left(F\right)\right)\right)=\omega *\sigma \left(GAP\left(F\right)\right)$$

In Equation (4), $EM_{c}$ stands for Efficient Channel Attention Mechanism, *F* is the original input feature, ω is the shared parameter, and σ denotes the sigmoid function.

In contrast to the ECAM, the SAM implements a compression of the channel dimension while keeping the spatial dimension constant. It applies maximum pooling and average pooling to the output from ECAM to obtain two $\begin{array}{c} 1\times H\times W \end{array}$ feature maps. Next, the two feature maps were concatenated and altered by $\begin{array}{c} 7\times 7 \end{array}$ convolution for 1 channel. The spatial attention feature maps are obtained using sigmoid, and finally, the output is multiplied by the original feature maps to return the size of $\begin{array}{c} C\times H\times W \end{array}$.

(5) $$\begin{array}{cc} M_{s}\left(F\right) & =\sigma (f^{7\times 7}\left(\left[A\nu gPool\left(F\right);MaxPool\left(F\right)\right]\right))\\ \; & =\sigma (f^{7\times 7}\left(\left[F_{a\nu g}^{s};F_{\max}^{s}\right]\right)) \end{array}$$

The $M_{s}$ represents spatial attention mapping, and $F_{a\nu g}^{s}$ and $F_{\max}^{s}$ represent average and max pooling.

**Figure 1:**
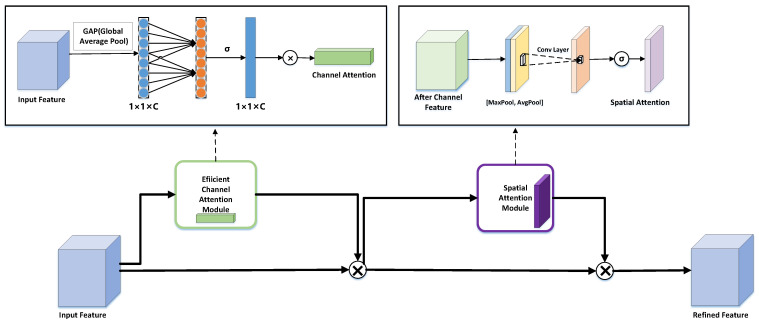
Model diagram of the ECBAM attention mechanism.

The ECBAM changes the architecture of the original CAM, where the main body ECAM learns channel information by using $\begin{array}{c} 1\times 1 \end{array}$ dynamic convolution and also uses an effective dimensionless approach to avoid dimensionality reduction while learning channel concern information, which improves the network performance. The ECBAM can be roughly expressed by Equation (6), and Figure 1 shows the structure of the ECBAM model.

(6) $$\left\{\begin{array}{c} F^{\prime }=EM_{c}⊗F\\ F^{\prime \prime }=M_{cs}⊗F^{\prime } \end{array}\right.$$

### 3.4. ECBAM_ASPP Model

Atrous Spatial Pyramid Pooling (ASPP) samples the input features at different sampling rates, obtains multi-scale feature information, and fuses it to obtain the final feature map.

In the previous subsection, we proposed an attention mechanism ECBAM. Based on this, we constructed a novel lane detection model ECBAM_ASPP in this section. The ASPP and ECBAM are the two main modules of the model. The raw feature images are first processed by the ASPP module and the results are used as the initial input to the ECBAM model. That is, the weight values generated by ECBAM are multiplied by the output of the ASPP module as input features, and the subsequent processing of the ECBAM module is performed sequentially.

**Figure 2:**
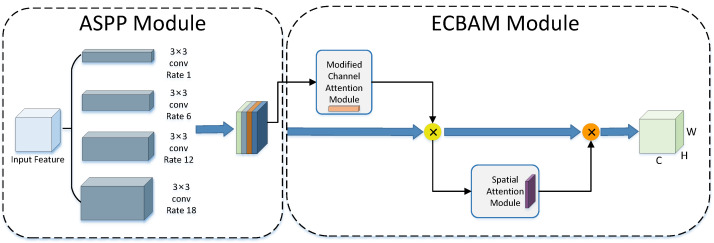
Framework diagram of the ECBAM_ASPP model.

The ECBAM_ASPP model first extracts input feature maps using the ASPP mechanism with sampling rates of 1, 6, 12, and 18. The extraction of multiple scales of information is performed by setting different sampling rates to capture sensory fields at different scales. Note that the size of the convolution kernel will remain $\begin{array}{c} 3\times 3 \end{array}$ throughout the entire process. The results extracted by ASPP are fed into the ECBAM module and first convolved in 1*D* by ECAM. To obtain the correlation feature map, the extracted feature maps from ASPP are multiplied by the results. The output of this stage is the input to the SAM to perform a 2*D* convolution of the Spatial Attention Module. Its output multiplies the feature maps obtained from the ECAM module to obtain the final output feature maps. Figure 2 shows the ECBAM_ASPP model.

### 3.5. Lane Detection

**Figure 3:**
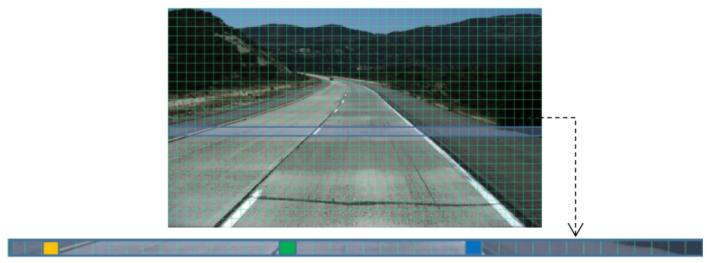
Lane representation based on row selection classification, with yellow, green, and blue squares representing lane line markers.

The lane lines are characterized as long and narrow, and the lane pixels make up a much smaller percentage of the image than the background pixels. To address this characteristic, this paper refers to the idea of line anchor classification proposed by UFLD [18], which defines lane detection as the selection and classification of horizontal positions in the line direction. This turns lane detection into a problem of selecting a particular grid over predefined row anchor boxes. Figure 3 below illustrates the general idea.

**Figure 4:**
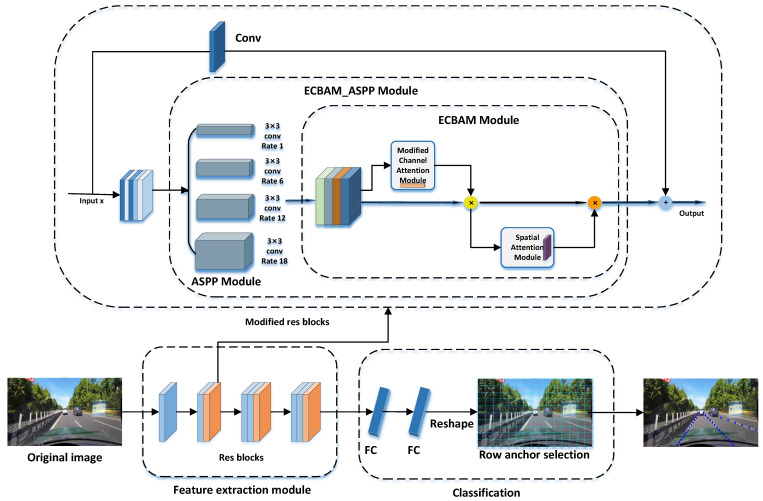
Overall model frame diagram.

Based on the concept of row-selective classification, we reconstruct the image into a feature map with a channel dimension of $C^{\prime }$ is 4, representing the detection of up to four lanes. Each channel’s feature map is divided into *h* rows, and each row is further split vertically into *w* grids, effectively partitioning the image of size $H_{0}\times W_{0}$ into h×w cells. To handle cases where no lanes exist, we add an additional (w+1)−th grid cell to the far-right of the feature map, indicating the absence of lanes in a given row. Following the approach of UFLD, classification is performed along the row direction, selecting the grid cells containing lane line elements from the w+1 grid cells. Instead of pixel-by-pixel classification, our model employs grid-based lane position selection, predicting lane-containing pixels while significantly improving detection speed. Figure 4 shows the overall model framework of lane detection in this paper.

## 4. Experiments

To evaluate the robustness and accuracy of the ECBAM_ASPP model, we compare and analyze its performance against several deep learning baseline methods on two datasets. The experimental results are further examined to demonstrate the effectiveness of the proposed model.

### 4.1. Datasets

To evaluate the proposed method, we conducted experiments on two widely recognized benchmark datasets: the TuSimple dataset [29] and the CULane dataset [30]. These datasets are considered standard benchmarks in the field of lane detection. Detailed information about the datasets is provided in Table 1.

**Figure 5:**
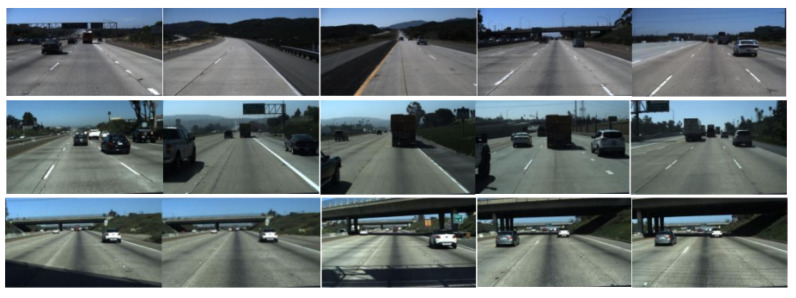
Some sample images from the TuSimple dataset.

The Tusimple dataset contains 3626 training samples, 358 validation samples, and 2782 test samples, each of which contains 20 consecutive frames captured per second. It is worth noting that only the 20th frame in each sequence is annotated with real lane markings. The driving scenes include various road conditions, such as intersections and curves, with 2 to 5 lanes annotated per frame. This dataset focuses on highway environments under clear weather conditions, where lane lines are represented by dotted line annotations. Figure 5 provides examples of images from this dataset.

**Figure 6:**
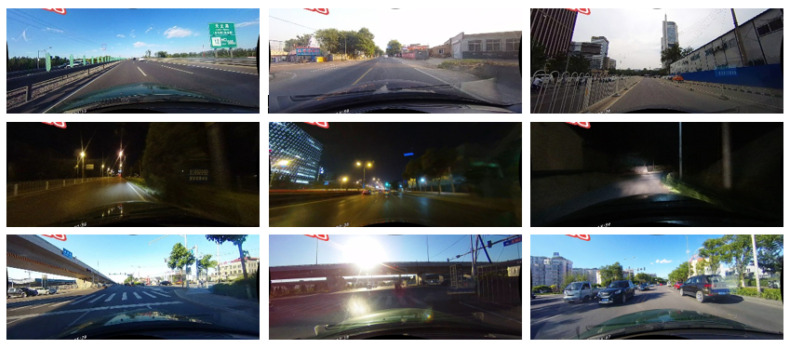
Some sample images from the CULane dataset.

The CULane dataset consists of 88,880 training samples, 9675 validation samples, and 34,680 test samples. It encompasses nine distinct scenarios, including normal, crowd, curve, glare, night, wireless, shadow, arrow, and urban scenes. The dataset was collected using six taxis in Beijing, with up to four lane lines annotated per image. Figure 6 presents example images from the dataset.

### 4.2. Experimental Environment and Details

Experimental Environment: The experiments were conducted on a computer equipped with an AMD Ryzen 7 5800H processor (3.20 GHz), Radeon Graphics, 16 GB of RAM, and an RTX 3050 graphics card. Additionally, a cloud server with an RTX 3090 graphics card was utilized to support the computations.

Implementation Details: To evaluate the effectiveness of the proposed model, we utilized a 34-layer ResNet [31] as the backbone. For training on the TuSimple dataset, input images were resized to 480×640, and 100 grids were used for row-based classification. For training on the CULane dataset, input images were resized to 288×800, with 150 grids employed for row-based classification. The Adam optimizer was used to optimize network parameters, with a cosine decay learning rate strategy. The initial learning rate was set to 0.0001, weight decay to $1\times 10^{-4}$, and momentum to 0.9. A batch size of 32 was used, with the model trained for 100 epochs on the TuSimple dataset and 50 epochs on the CULane dataset.

### 4.3. Evaluation Metrics

On the TuSimple dataset, we use accuracy and F1-score to evaluate the lane line detection performance. Accuracy represents the ratio of correctly predicted pixels to the total number of pixels in a sample. The F1-score is calculated to assess the balance between precision and recall, distinguishing whether a prediction is a true positive (TP), false positive (FP), or false negative (FN) case. This distinction is determined based on the Intersection over Union (IoU) between the predicted lane line and the actual ground truth label. The accuracy and F1-score are computed using the following formulas:

(7) $$\mathrm{Accuracy}=\frac{True\;Positive+True\;Negative}{Total\;Number\;of\;Pixels}$$

(8) $$\mathrm{precision}\;=\frac{TP}{TP+FP},\;\mathrm{recall}=\frac{TP}{TP+FN}$$

(9) $$F1=\frac{2\times precision\times recall}{precision+recall}$$

On the CULane dataset, the F1-score is selected as the evaluation metric, with its calculation defined in Equation (9). Additionally, for both datasets, the frame rate (FPS) is used to assess the real-time performance of the proposed model. Real-time performance is a critical metric in autonomous driving, as it directly impacts the responsiveness and feedback speed of vehicle control.

### 4.4. Comparative Analysis of Experiments

#### 4.4.1. Tusimple Dataset

To evaluate the detection performance of the proposed model on the TuSimple dataset, we compared the ECBAM_ASPP model with nine baseline methods: PolyLaneNet [32], FastDraw [33], PINet [34], EL-GAN [35], SAD [1], SCNN [16], UFLD [18], RATS [23], and MKD [21]. Table 2 presents the evaluation results on the TuSimple dataset.

**Figure 7:**
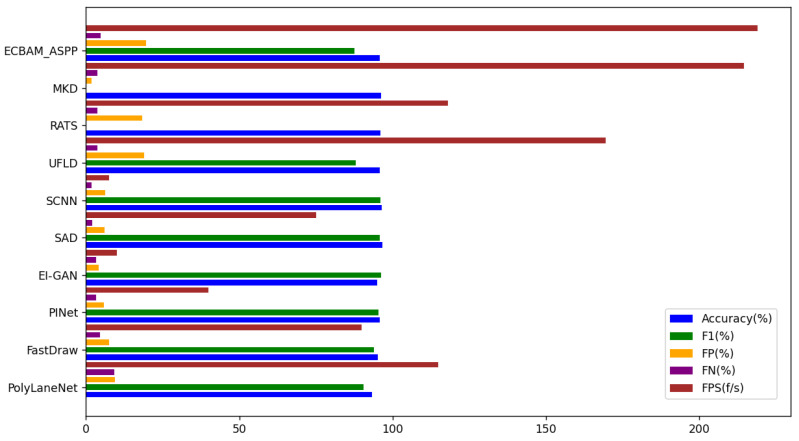
The visual results for each model.

The visualization results in Figure 7 provide a clear and intuitive comparison of the performance of each model. In the figure, the F1-score for RATS and MKD is 0, indicating that no accuracy measurement is available for these methods. Compared with approaches that rely on semantic segmentation and clustering instances, our pixel block classification model significantly reduces computational costs. This reduction not only improves detection accuracy but also achieves a fast detection speed, averaging 219 frames per second. As shown in Table 2, while the detection accuracy of our model is approximately 0.75% lower than that of SCNN, its detection speed is nearly 29 times faster. These results demonstrate that the ECBAM_ASPP model achieves substantial real-time performance improvements while maintaining high accuracy, making it well suited for task scenarios with stringent real-time requirements, such as autonomous driving.

**Figure 8:**
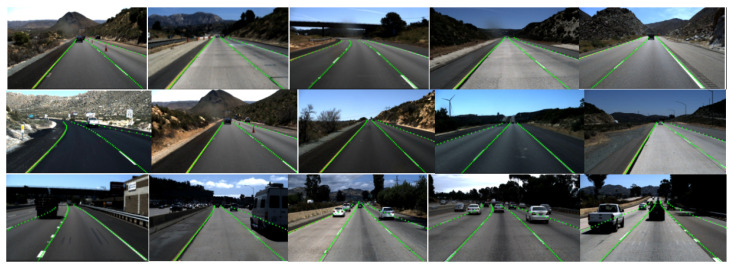
Detection results of the ECBAM_ASPP on TuSimple dataset.

Figure 8 shows the test results of the proposed ECBAM_ASPP lane detection model on the TuSimple dataset. The prediction of straight lanes is basically accurate; however, minor deviations are observed near the vanishing points of curved lanes, especially near the endpoints. This indicates that the model does not fully capture non-local information and misses some finer details. Although these deviations have a limited impact on the overall accuracy, they may pose risks in some high-speed scenarios, highlighting the need for further optimization.

#### 4.4.2. CULane Dataset

To evaluate the detection performance of the proposed model on the CULane dataset, we compared the ECBAM_ASPP model with eight baseline methods: SCNN [16], SAD [1], UFLD [18], LaneATT [36], CondLaneNet [5], GANet [6], O2SFormer [37], MKD [21]. Table 3 presents the evaluation results on the CULane dataset.

Table 3 presents the evaluation results on the CULane dataset. The F1-score is used as the evaluation metric for the overall scene and eight subscenes, excluding the “intersection” scene. For the intersection scene, false positives (FP) are used as the evaluation metric, as no lanes are present in this scenario. As shown in Table 3, our model achieves an F1-score of 94.08% in the normal scene. Although the F1-scores in other scenes are slightly lower than those of some of the latest methods, its frame rate (FPS) reaches 227.3. This demonstrates that the ECBAM_ASPP model outperforms other baseline methods in terms of real-time performance.

**Figure 9:**
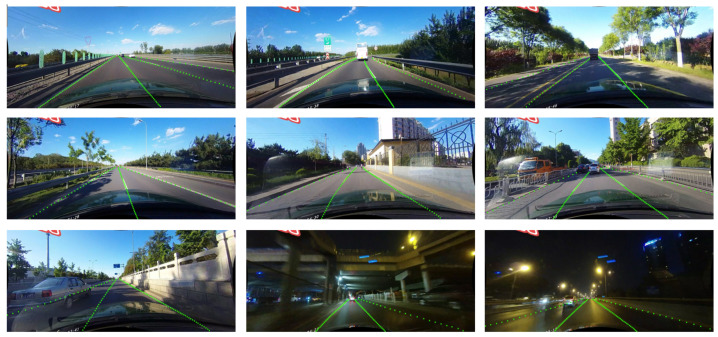
Detection results of the ECBAM_ASPP on CULane dataset.

Figure 9 presents the test results of the proposed ECBAM_ASPP lane detection model on the CULane dataset. While the model demonstrates excellent real-time performance, its F1-score is relatively low in certain scenarios, highlighting limitations in lane detection capability, particularly in dazzle lights, no line, and cross road.

### 4.5. Ablation Experiments

We conducted experiments on the TuSimple dataset to evaluate the effectiveness of different components in the ECBAM_ASPP model. Using the same experimental parameter settings, we validated four scenarios: without the ASPP module or the ECBAM attention mechanism, with the ECBAM attention mechanism but without the ASPP module, with the ASPP module but without the ECBAM attention mechanism, and with both the ASPP module and the ECBAM attention mechanism. The experimental results are summarized in Table 4.

As shown in Table 4, incorporating either the ASPP module or the ECBAM module individually results in a moderate improvement in lane detection accuracy. However, the ECBAM_ASPP model, which integrates the ECBAM module and incorporates the ASPP module, achieves a significant enhancement in lane detection performance. This indicates that the ECBAM attention mechanism effectively focuses the model’s attention on regions of interest while suppressing irrelevant information, and the ASPP mechanism enhances the richness of features extracted by the model. In conclusion, the ECBAM_ASPP model demonstrates accurate, real-time lane detection with strong robustness.

We conducted an ablation experiment in which the CBAM module was replaced by the ECBAM module to assess its effectiveness and significance. The results, presented in Table 5, indicate that the model achieves higher detection accuracy when the ECBAM attention mechanism proposed in this paper is used compared with CBAM. These findings demonstrate that the ECBAM attention mechanism contributes to improving the model’s accuracy, highlighting its importance in lane detection tasks.

## 5. Conclusions

The primary objective of this paper is to design a model that enhances both the accuracy and speed of lane detection. To achieve this, we propose the ECBAM_ASPP model, which is based on the concept of line-anchor classification. By integrating the ASPP and ECBAM attention mechanisms, the model effectively and accurately detects lane lines while significantly improving detection speed. The ECBAM attention mechanism introduced in this paper preserves the advantages of the original CBAM mechanism while addressing its limitations related to dimensionality reduction. It suppresses irrelevant information, strengthens the focus on lane-related features, and enhances the effectiveness of the channel attention mechanism, thereby improving overall model performance. Experimental results on the TuSimple and CULane datasets demonstrate that the proposed model achieves substantial improvements in real-time performance while maintaining competitive accuracy compared with baseline methods.

Although our model demonstrates fast lane detection, it still has certain limitations. For instance, its accuracy can decrease in challenging scenarios such as extreme glare, severe occlusion, or unclear lane markings. Additionally, in curved lane scenarios, the model may struggle to fully capture non-local information, particularly when a broader spatial context is needed. In future work, we will focus on enhancing the accuracy and efficiency of lane detection in these challenging conditions. 

## Figures and Tables

**Table 1:** Details of the two lane detection datasets.

Dataset	Frame	Train	Validation	Test	Lane	Resolution	Scenarios
Tusimple	6408	3268	358	2782	≤5	$\begin{array}{c} 1280\times 720 \end{array}$	1
CULane	133,235	88,880	9675	34,680	≤4	$\begin{array}{c} 1640\times 590 \end{array}$	9

**Table 2:** Results for each model in the TuSimple dataset.

Method Name	Accuracy (%)	F1 (%)	FP (%)	FN (%)	FPS (f/s)
PolyLaneNet	93.36	90.62	9.42	9.33	115
FastDraw	95.2	93.92	7.6	4.5	90
PINet	95.81	95.39	5.85	3.36	40
EL-GAN	94.9	**96.26**	4.12	3.36	10
SAD	**96.64**	95.92	6.02	2.05	75
SCNN	96.53	95.97	6.17	**1.8**	7.5
UFLD	95.86	88.02	18.91	3.75	169.5
RATS	96.16	-	18.3	3.62	118
MKD	96.27	-	**1.79**	3.8	214.6
ECBAM_ASPP	95.78	87.67	19.7	4.7	**219**

**Table 3:** Results for each model in the CULane dataset.

Method Name	Total	Normal	Crowded	Dazzle	Shawdow	No line	Arrow	Curve	Cross	Night	FPS
SCNN	71.6	90.6	69.7	58.5	66.9	43.4	84.1	64.4	1990	66.1	7.5
SAD	70.7	89.9	68.5	59.9	67.7	42.2	83.8	66.0	1960	64.6	19.8
UFLD	72.3	90.7	70.2	59.5	69.3	44.4	85.7	69.5	2037	66.7	175.4
LaneATT	76.68	92.14	75.03	66.47	78.15	49.39	88.38	67.72	**1330**	70.72	129
CondLaneNet	78.74	93.38	77.14	71.17	79.93	51.85	89.89	73.88	1387	73.92	128
GANet	79.39	93.73	77.92	**71.64**	79.49	52.63	**90.37**	76.32	1368	73.67	127
O2SFormer	77.03	92.5	75.25	70.93	77.72	50.97	87.63	68.1	2749	72.88	-
MKD	**79.69**	93.62	**78.93**	61.41	**80.56**	**61.81**	88.58	**76.56**	3102	**76.36**	214.8
ECBAM_ASPP	78.45	**94.08**	77.17	68.56	79.20	51.18	88.72	75.41	2559	73.71	**227.3**

**Table 4:** Detection Results For Each Model in the TuSimple Dataset.

Group Number	ASPP Module	ECBAM Module	Accuracy (%)
1			95.06
2	✓		95.35
3		✓	95.48
4	✓	✓	**95.78**

**Table 5:** The Results of the ECABM Ablation Experiments.

Group Number	CBAM Module	ECBAM Module	Accuracy (%)
1			95.35
2	✓		95.42
3		✓	**95.78**

## Data Availability

The data analyzed during the current study are available from the corresponding author upon reasonable request.

## Abbreviations

The following abbreviations are used in this paper:

ECBAM	Efficient Convolution Block Attention Module
CBAM	Convolutional Block Attention Module
ASPP	Atrous Spatial Pyramid Pooling
ECBAM_ASPP	Efficient Convolution Block Attention Module_Atrous Spatial Pyramid Pooling
ECA	Efficient Channel Attention
